# A Study of the Polarization of Fluorescence of Ordered Systems With Application to Ordered Liquid Crystals

**DOI:** 10.6028/jres.080A.005

**Published:** 1976-02-01

**Authors:** E. D. Cehelnik, K. D. Mielenz, R. B. Cundall

**Affiliations:** Institute for Materials Research, National Bureau of Standards, Washington, D.C. 20234; Chemistry Department, The University of Nottingham, Nottington, NG72RD, England

**Keywords:** 1,6-Diphenyl-1,3,5-hexatriene, fluorescence, linear dichroism, liquid crystals, polarization, spectrofluorimetry

## Abstract

The fluorescence polarization of uniaxial molecules dissolved in an ordered medium is studied. A theoretical model is developed which relates the polarization of the fluorescence emission to molecular structure, orientation of absorption and emission dipole oscillators and the degree of ordering. This theory was tested experimentally using all trans 1,6-diphenyl-1,3,5-hexatriene dissolved in an ordered liquid crystal.

## 1. Introduction

In continuation of previous polarization studies of ordered liquid crystal [1, 2]^1^ and isotropic liquid systems [2, 3, 4], this work is concerned with the polarization of fluorescence by molecules dissolved in an ordered liquid crystal. A general theoretical model is developed that can be used with various linearly dichroic systems, such as streaming solutions or stretched polymer films, molecular crystals or membranes. The model is verified experimentally for the case of a fluorescent dye dissolved in an ordered nematic liquid crystal.

The liquid crystal solvent used here was a mixture of cholesteryl chloride and cholesteryl laurate [5, 2, 1]. These mixtures are normally cholesteric with a helical arrangement of the molecules. However, at a definite composition and temperature, *T*_nem_, a nematic state results [5]. In the “unordered” nematic state of the mesophase the molecules are parallel within domaines. These domaines are oriented randomly with respect to each other thus giving the characteristic cloudiness and opacity of nematic liquid crystals. When an electric field is applied the domaines orient in such a way that the solvent molecules are parallel to the electric field [5].

The liquid crystal mixture (L.C.M.) changes from an opaque to a clear state within a few seconds, this change being reversible once the field is turned off. This ordering phenomenon can be used to order solute molecules which are dissolved in the L.C.M. at low concentrations. The L.C.M. used was a 1.95/1 by weight ratio of cholesteryl chloride to cholesteryl laurate, which is nematic at *T*_nem_ = 30 °C [5, 2, 1]. Ordering was achieved by application of an electric field of the order of 40 kV/cm.

The solute molecule used here as well as previously [1, 2] is 1,6-diphenyl-1,3,5-hexatriene (DPH), which is a long “rod like” molecule that absorbs light in the ultraviolet and fluoresces with a blue emission. DPH is fairly soluble in this L.C.M. and is aligned with its long molecular axis parallel to the direction of orientation of the ordered liquid crystal [1, 2].

## 2. Theory

### 2.1. Definitions and Outline of Approach

**Figure 1:**
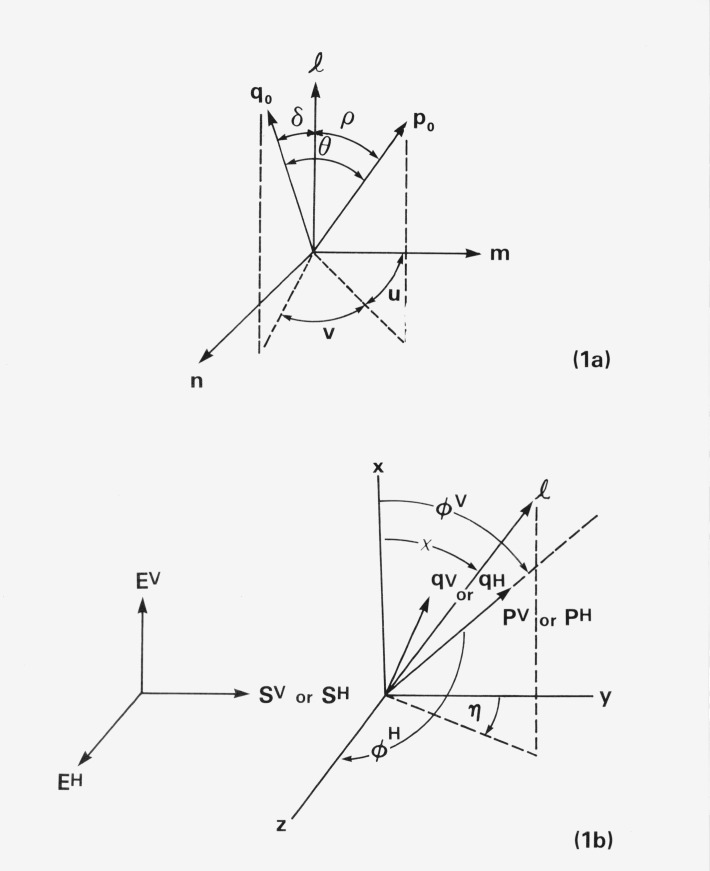
Diagrams of the model showing the molecular reference frame (1a) and the laboratory reference frame (1b) where *p* refers to the absorption dipole, *q* refers to the emission dipole and *1* refers to the long molecular axis of the molecule.

As a theoretical model for the fluorescence polarization of an ordered sample, we consider a collection of uniaxial solute molecules with absorption and emission dipole oscillators that have a fixed orientation with respect to the molecular axis as indicated in figure 1a. This fixed orientation is defined by three angles: *ρ* and δ, the angles between the unit vector in the direction of the molecular axis, **1**, and the unit vectors in the directions of the absorption and emission dipole oscillators, **p**_0_ and **q**_0_, respectively; and *v*, the angle enclosed by the planes (**1, p**_0_) and (**1, q**_0_). Thus we have, relative to a molecular reference frame consisting of **1** and two arbitrarily chosen orthogonal unit vectors **m** and **n** in the plane perpendicular to **1**,

$$p_{0}=\left[\cos\;\rho ,\sin\;\rho \;\cos\;\left(u\right),\sin\;\rho \;\sin\;\left(u\right)\right],$$ (1a)

$$q_{0}=\left[\cos\;\delta ,\sin\;\delta \;\cos\;\left(u+v\right),\sin\;\delta \;\sin\;\left(u+v\right)\right],$$ (1b)

where *u* is defined in figure 1a. A further angle defined by the molecular structure is *θ*, the angle between the absorption and emission dipole oscillators. According to eqs (1a, b), it is related to *ρ*, δ, and *v* by

$$\begin{array}{l} \cos\;\theta =p_{0}\cdot q_{0}\\ \;=\cos\;\rho \;\cos\;\delta +\sin\;\rho \;\sin\;\delta \;\cos\;\left(v\right). \end{array}$$ (1c)

It will be assumed that each molecule can rotate about its molecular axis. Then, different molecules have rotated through different angles *u* at the time of excitation so that, for the sample as a whole, *u* is a random variable. The angle *v* that enters into the theory depends on the relative orientation of the absorption dipole oscillator **p**_0_ *at the time of absorption* with respect to the emission dipole oscillator **q**_0_ *at the time of emission.* Therefore, *v* becomes equal to the intramolecular angle shown in figure 1a in the borderline case of molecules that do not rotate appreciably during the time interval between absorption and emission, but becomes a random variable for a large number of molecules that rotate fast and have long fluorescence lifetimes. The theory presented here treats the general case in which *v* falls anywhere within this range.

The ordering of the molecules is produced by an external force, such as an electric or magnetic field, that is applied to the sample. It is assumed that this force hinders the tumbling motion of the individual molecular axes so that, for each molecule, the angle χ between the molecular axis and the direction of the applied force does not change appreciably between the times of absorption and emission. The applied force produces a preferential orientation of the molecular axes with respect to the *x* axis of the laboratory reference frame in figure 1b. This alinement of the molecules is defined by a distribution function *f*(χ), so that *f*(χ) sin χ*d*χ is proportional to the number of molecules with molecular axes that enclose angles between χ and χ + *d*χ with the *x* axis. The angular distribution of the molecular axes is assumed to be symmetric about the *x* axis, so that *f*(−χ) = *f*(χ); and it is also assumed that the probabilities for “upside-up” and “upside-down” alinement of a molecule are the same, so that *f*(χ + π) *= f*(χ). A suitable form for *f*(χ) is the quasi-gaussian distribution function used in an earlier paper [1],

$$f\left(\chi \right)=e^{\kappa \left(|\cos\;\chi |-1\right)},$$ (2a)

where *e^κ^ = f*(0)/*f*(π) defines the proportion of molecules oriented parallel and perpendicular to the *x* axis, respectively. This function *f*(χ) leads to the following closed expressions for the averages of cos^2^χ and cos^4^χ that will appear in the equations to be developed:

$$N\equiv \bar{\cos^{2}\chi }\equiv \frac{\int_{-\pi }^{\pi }\cos^{2}\chi f\left(\chi \right)\;\sin\;\chi d\chi }{\int_{-\pi }^{\pi }f\left(\chi \right)\;\sin\;\chi d\chi }=\frac{\kappa^{2}-2\kappa +2\left(1-e^{-\kappa }\right)}{\kappa^{2}\left(1-e^{-\kappa }\right)},$$ (2b)

$$M\equiv \bar{\cos^{4}\chi }\equiv \frac{\int_{-\pi }^{\pi }\cos^{4}\chi f\left(\chi \right)\;\sin\;\chi d\chi }{\int_{-\pi }^{\pi }f\left(\chi \right)\;\sin\;\chi d\chi }=\frac{\kappa^{4}-4\kappa^{3}+12\kappa^{2}-24\kappa +24\left(1-e^{-\kappa }\right)}{\kappa^{4}\left(1-e^{-\kappa }\right)}.$$ (2c)

**Figure 2:**
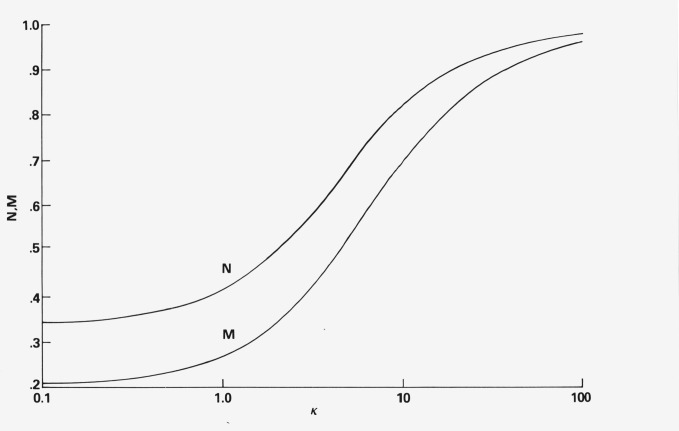
Calculated plots of the two orientation terms *N* and *M* as a function of one adjustable parameter κ where the distribution function is assumed to be of the form, *f*(χ) = *e*^κ(|cos χ |−1)^.

A plot of *N* and *M* versus *κ* is reproduced in figure 2. The limits of *N* are 1/3 for a random distribution of absorbing molecules (*f*(χ) ≡ 1) and 1.0 for perfect ordering (*f*(χ) ≡ Dirac delta function). The corresponding limits for ***M*** are 1/5 and 1.0 respectively. The transition between these two borderline cases depends on the specific form of *f*(χ).

As also indicated in figure 1b, the exciting radiation is assumed to be incident upon the sample in the *y*-direction of the laboratory reference frame, and is assumed to be plane polarized with its electric field vector, **E***^V^* or **E***^H^*, parallel to the *x* or *z* axes. The corresponding photon flux densities of the exciting radiation are denoted by *S^V^* and *S^H^*, respectively. The squared magnitudes of the absorption and emission dipole vectors **p** and **q** are taken to be equal to the probabilities for absorption and emission by a molecule, so that for excitation with vertically polarized radiation,

$${\left|p^{V}\right|}^{2}=S^{V}\sigma_{0}\cos^{2}\phi^{V},\;{\left|q^{V}\right|}^{2}=Q{\left|p^{V}\right|}^{2},$$ (3a)

and for excitation with horizontally polarized radiation,

$${\left|p^{H}\right|}^{2}=S^{H}\sigma_{0}\cos^{2}\phi^{H},\;{\left|q^{H}\right|}^{2}=Q{\left|p^{H}\right|}^{2},$$ (3b)

where the superscripts *V* and *H* denote the polarization of the exciting radiation. Here, *ϕ^V^* and *ϕ^H^* are the respective angles between the absorption oscillator and the exciting electric field, σ_0_ is the absorption cross section for absorption oscillators that are parallel to the exciting field, and *Q* is the fluorescence quantum yield.

The components of the, absorption and emission dipole vectors with respect to the laboratory axes can now be calculated as follows. According to figure 1b we have

l=(cosχ,sinχcosη,sinχsinη), (4a)

where *η* is a random angle because of the rotation of the molecule about the molecular axis. Then, the two unit vectors

$$\begin{array}{l} m=\left(-\sin\;\chi ,\cos\;\chi \;\cos\;\eta ,\cos\;\chi \;\sin\;\eta \right),\\ n=\left(0,-\sin\;\eta ,\cos\;\eta \right) \end{array}$$ (4b)

(not shown in figure 1b) may be taken to represent the other two axes of the molecular reference frame, so that

$$A=\left(\begin{array}{lll} \cos\;\chi & -\sin\;\chi & 0\\ \sin\;\chi \;\cos\;\eta & \cos\;\chi \;\cos\;\eta & -\sin\;\eta \\ \sin\;\chi \;\sin\;\eta & \cos\;\chi \;\sin\;\eta & \cos\;\eta \end{array}\right)$$ (4d)

is a suitable matrix to transform molecular coordinates into laboratory coordinates. Hence, eqs (1a) and (4d) show that the *x* and *z* components of the unit vector in the direction of the absorption dipole oscillator **p** in figure 1b are

$${\left(Ap_{0}\right)}_{x}\equiv \cos\phi^{V}=\cos\;\chi \;\cos\;\rho -\sin\;\chi \;\sin\;\rho \;\cos\;\left(u\right),$$ (5a)

$${\left(Ap_{0}\right)}_{z}\equiv \cos\phi^{H}=\sin\;\chi \;\sin\;\eta \;\cos\;\rho +\cos\;\chi \;\sin\;\eta \;\cos\;\left(u\right)+\cos\;\eta \;\sin\;\left(u\right)\;\sin\;\rho .$$ (5b)

Similarly, eqs (1b) and (4d) yield the unit vector **Aq**_0_ in the direction of the emission dipole oscillator **q**, so that

$$q^{V}=\sqrt{\sigma_{0}QS^{V}}{\left(Ap_{0}\right)}_{x}Aq_{0},$$ (6a)

$$q^{H}=\sqrt{\sigma_{0}QS^{H}}{\left(Ap_{0}\right)}_{z}Aq_{0},$$ (6b)

according to eqs (3a, b) and (6a, b). Here,

$${\left(Aq_{0}\right)}_{x}=\cos\;\chi \;\cos\;\delta -\sin\;\chi \;\sin\;\delta \;\cos\;\left(u+v\right),$$ (7a)

$${\left(Aq_{0}\right)}_{y}=\sin\;\chi \;\cos\;\eta \;\cos\;\delta +\left[\cos\;\chi \;\cos\;\eta \;\cos\;\left(u+v\right)-\sin\;\eta \;\sin\;\left(u+v\right)\right]\;\sin\;\delta ,$$ (7b)

$${\left(Aq_{0}\right)}_{z}=\sin\;\chi \;\sin\;\eta \;\cos\;\delta +\left[\cos\;\chi \;\sin\;\eta \;\cos\;\left(u+v\right)+\cos\;\eta \;\sin\;\left(u+v\right)\right]\;\sin\;\delta .$$ (7c)

In order to describe the sample as a whole, these expressions must be replaced by their rms averages for a large number of molecules.

The fluorescence intensity *I* emitted by the sample into a given viewing direction is proportional to the squared rms components of **q***^V^* and **q***^H^* in the plane perpendicular to the viewing direction, and is polarized in the directions of these components. Therefore, the four intensity readings that are obtained for the four possible combinations of vertical and horizontal setting of a pair of excitation and emission polarizers are, for 90° viewing in the direction of the *z* axis,

$$R_{V}^{V}\left(90{}^{\circ}\right)=T_{V}k\bar{{\left(q_{x}^{V}\right)}^{2}},R_{H}^{V}\left(90{}^{\circ}\right)=T_{H}k\bar{{\left(q_{y}^{V}\right)}^{2}},$$ (8a)

$$R_{V}^{H}\left(90{}^{\circ}\right)=T_{V}k\bar{{\left(q_{x}^{H}\right)}^{2}},R_{H}^{H}\left(90{}^{\circ}\right)=T_{H}k\bar{{\left(q_{y}^{H}\right)}^{2}},$$ (8b)

and for 180° viewing in the direction of the *y* axis,

$$R_{V}^{V}\left(180{}^{\circ}\right)=T_{V}k\bar{{\left(q_{x}^{V}\right)}^{2}},R_{H}^{V}\left(180{}^{\circ}\right)=T_{H}k\bar{{\left(q_{z}^{V}\right)}^{2}},$$ (8c)

$$R_{V}^{H}\left(180{}^{\circ}\right)=T_{V}k\bar{{\left(q_{x}^{H}\right)}^{2}},R_{H}^{H}\left(180{}^{\circ}\right)=T_{H}k\bar{{\left(q_{z}^{H}\right)}^{2}},$$ (8d)

Here, superscripts and subscripts denote the respective settings of the excitation and emission polarizers, *T_V_* and *T_H_* are the responsivities of the emission detection system for vertically and horizontally polarized light, and *k* is a geometry factor.

At any other viewing angle, the readings obtained can be expressed as linear combinations of the 90° and 180° readings. For example, for an arbitrary viewing angle *α*, the reading with both polarizers in the horizontal position is

$$\begin{array}{l} R_{H}^{H}\left(\alpha \right)=R_{H}^{H}\left(90{}^{\circ}\right)\;\sin^{2}\left(\alpha \right)+R_{H}^{H}\left(180{}^{\circ}\right)\;\cos^{2}\left(\alpha \right)\\ \;=T_{H}k\left[{\left(q_{y}^{H}\right)}^{2}\sin^{2}\left(\alpha \right)+{\left(q_{z}^{H}\right)}^{2}\cos^{2}\left(\alpha \right)\right]. \end{array}$$ (8e)

### 2.2. Derivation of Equations

The six vector coordinates that completely define the system are found by combining eqs (5a) and (5b) with eqs (7a), (7b), and (7c) to give

$${\left(q_{x}^{V}\right)}^{2}=S^{V}\sigma_{0}Q{\left(Ap_{0}\right)}_{x}\;{\left(Aq_{0}\right)}_{x}$$ (9a)

$${\left(q_{y}^{V}\right)}^{2}=S^{V}\sigma_{0}Q{\left(Ap_{0}\right)}_{x}\;{\left(Aq_{0}\right)}_{y}$$ (9b)

$${\left(q_{z}^{V}\right)}^{2}=S^{V}\sigma_{0}Q{\left(Ap_{0}\right)}_{x}\;{\left(Aq_{0}\right)}_{z}$$ (9c)

$${\left(q_{x}^{H}\right)}^{2}=S^{H}\sigma_{0}Q{\left(Ap_{0}\right)}_{z}\;{\left(Aq_{0}\right)}_{x}$$ (9d)

$${\left(q_{y}^{H}\right)}^{2}=S^{H}\sigma_{0}Q{\left(Ap_{0}\right)}_{z}\;{\left(Aq_{0}\right)}_{y}$$ (9e)

$${\left(q_{z}^{H}\right)}^{2}=S^{H}\sigma_{0}Q{\left(Ap_{0}\right)}_{z}\;{\left(Aq_{0}\right)}_{y}.$$ (9f)

In this work, the averaging of these expressions was done in three steps. First the terms were multiplied through and averaged with respect to all possible angles *u* using

$$\begin{array}{c} \frac{\int_{0}^{2\pi }\cos\;udu}{\int_{0}^{2\pi }du}=0,\;\frac{\int_{0}^{2\pi }\cos{\;}^{2}udu}{\int_{0}^{2\pi }du}=\frac{1}{2}\\ \frac{\int_{0}^{2\pi }\cos^{3}\;udu}{\int_{0}^{2\pi }du}=0,\;\frac{\int_{0}^{2\pi }\cos{\;}^{4}udu}{\int_{0}^{2\pi }du}=\frac{3}{8}, \end{array}$$ (10)

*u* being a random variable. Next they were averaged over all possible angles *η*, using analogous equations to eqs (10) since *η* was also assumed to be a random variable. Finally the terms were collected and expressed as functions of *N* and *M* as given by eqs (2b) and (2c). The resultant expressions were found to be

$${\left(\bar{q_{x}^{V}}\right)}^{2}/S^{V}\sigma_{0}Q=\left(\frac{17M-10N+1}{8}\right)AB+\left(\frac{1-2N+M}{4}\right)C+2\left(N-M\right)D+\left(\frac{6N-5M-1}{8}\right)\left(A+B\right)+\left(\frac{1-2N+M}{4}\right),$$ (11a)

$${\left(q_{y}^{V}\right)}^{2}/S^{V}\sigma_{0}Q={\left(q_{z}^{V}\right)}^{2}/S^{V}\sigma_{0}Q=\left(\frac{10N-17M-1}{16}\right)AB+\left(\frac{2N-M-1}{8}\right)C+\left(M-N\right)D+\left(\frac{5M+6N-3}{16}\right)A+\left(\frac{5M-6N+1}{16}\right)B+\left(\frac{3-2N-M}{16}\right),$$ (11b, c)

$$\bar{{\left(q_{x}^{H}\right)}^{2}}/S^{H}\sigma_{0}Q=\left(\frac{10N-17M-1}{16}\right)AB+\left(\frac{2N-M-1}{8}\right)C+\left(M-N\right)D+\left(\frac{5M-6N+1}{16}\right)A+\left(\frac{5M+6N-3}{16}\right)B+\left(\frac{3-2N-M}{16}\right),$$ (11d)

$$\bar{{\left(q_{y}^{H}\right)}^{2}}/S^{H}\sigma_{0}Q=\left(\frac{17M+22N-15}{64}\right)AB+\left(\frac{M-10N+1}{32}\right)C+\left(\frac{2N-M-1}{4}\right)D+\left(\frac{11-30N-5M}{64}\right)\left(A+B\right)+\left(\frac{M+22N+1}{64}\right),$$ (11e)

$$\bar{{\left(q_{z}^{H}\right)}^{2}}/S^{H}\sigma_{0}Q=\left(\frac{51M-62N+19}{64}\right)AB+\left(\frac{3M+2N+3}{32}\right)C+\left(\frac{2N-3M+1}{4}\right)D+\left(\frac{6N-15M+1}{64}\right)\left(A+B\right)+\left(\frac{3M+2N+3}{64}\right);$$ (11f)

where

*A*: =cos^2^ *ρ*
*B*: =cos^2^ δ
*C*: =sin^2^ *ρ* sin^2^δ cos^2^ *v*
*D*: =cos *ρ* cos *δ* sin *ρ* sin *δ* cos *v*

The six general expressions can now be expressed in their final form of a contribution due to a dependence on *ρ* only and composite contributions of *ρ*, δ, and *v.*

These are

$$\bar{{\left(q_{x}^{V}\right)}^{2}}/S^{V}\sigma_{0}Q=\left(a^{V}+2b^{V}\right)$$ (12a)

$$\bar{{\left(q_{y}^{V}\right)}^{2}}/S^{V}\sigma_{0}Q={\left(q_{z}^{V}\right)}^{2}/S^{V}\sigma_{0}Q=\left(a^{V}-b^{V}\right)$$ (12b, c)

$$\bar{{\left(q_{x}^{H}\right)}^{2}}/S^{H}\sigma_{0}Q=\left(a^{H}-b^{H}+2c^{H}\right)$$ (12d)

$$\bar{{\left(q_{y}^{H}\right)}^{2}}/S^{H}\sigma_{0}Q=\left(a^{H}-b^{H}-c^{H}\right)$$ (12e)

$$\bar{{\left(q_{z}^{H}\right)}^{2}}/S^{H}\sigma_{0}Q=\left(a^{H}+2b^{H}-c^{H}\right)$$ (12f)

where

$$\begin{array}{l} a^{V}=\frac{1}{6}\left[N\left(3\;\cos^{2}\;\rho -1\right)+\sin^{2}\;\rho \right],\\ b^{V}=\frac{1}{24}\left[\left(9M-6N+1\right)\cos^{2}\;\rho -\left(3M-4N+1\right)\right]\left(3\;\cos^{2}\;\delta -1\right)\\ \;+\left(N-M\right)\cos\;\rho \;\cos\;\delta \;\sin\;\rho \;\sin\;\delta \;\cos\;v\\ \;+\frac{1}{16}\left(M-2N+1\right)\left(2\;\cos^{2}v-1\right)\;\sin^{2}\;\rho \;\sin^{2}\;\delta ,\\ a^{H}=\frac{1}{12}\left\{2-[\sin^{2}\;\rho +N\left(3\;\cos^{2}\;\rho -1\right)\right\},\\ b^{H}=\left(\frac{M-2N+1}{48}\right)\left(3\;\cos^{2}\;\delta -1\right)\left(3\;\cos^{2}\;\rho -1\right)\\ \;+\left(\frac{M-1}{6}\right)\;\cos\;\rho \;\cos\;\delta \;\sin\;\rho \;\sin\;\delta \;\cos\;v\\ \;+\left(\frac{M+6n+1}{96}\right)\left(2\;\cos^{2}v-1\right)\;\sin^{2}\;\rho \;\sin^{2}\;\delta ,\\ c^{H}=\frac{1}{96}\left[\left(5M+6N-3\right)-\left(15M-6N-1\right)\cos^{2}\;\rho \right]\left(3\;\cos^{2}\;\delta -1\right)\\ \;+\left(\frac{5M-6N+1}{12}\right)\cos\;\rho \;\cos\;\delta \;\sin\;\rho \;\sin\;\delta \;\cos\;v\\ \;+\left(\frac{18N-5M-5}{192}\right)\left(2\;\cos^{2}v-1\right)\;\sin^{2}\;\rho \;\sin^{2}\;\delta . \end{array}$$

The total intensities emitted by the sample for the two types of excitation, vertical and horizontal, are thus,

$$\begin{array}{c} \bar{{\left(q^{V}\right)}^{2}}/S^{V}\sigma_{0}Q=\left[\bar{{\left(q_{x}^{V}\right)}^{2}}+\bar{{\left(q_{y}^{V}\right)}^{2}}+\bar{{\left(q_{z}^{V}\right)}^{2}}\right]/S^{V}\sigma_{0}Q=3a^{V}\\ =\frac{N\left(3\;\cos^{2}\;\rho -1\right)+\sin^{2}\;\rho }{2} \end{array}$$ (13a)

$$\begin{array}{c} \bar{{\left(q^{H}\right)}^{2}}/S^{H}\sigma_{0}Q=\left[\bar{{\left(q_{x}^{H}\right)}^{2}}+\bar{{\left(q_{y}^{H}\right)}^{2}}+\bar{\left(q_{z}^{H}\right)}\right]/S^{V}\sigma_{0}Q=3a^{H}\\ =\frac{2-\sin^{2}\;\rho -N\left(3\;\cos^{2}\;\rho -1\right)}{4}. \end{array}$$ (13b)

Since the total intensity emitted by the sample is directly proportional to the total intensity absorbed the dichroic ratio for absorption can be given by

$$D_{a}=\bar{{\left(q^{V}\right)}^{2}}\bar{{{\;}^{/}q^{H})}^{2}}=\frac{S^{V}}{S^{H}}\frac{2N+\left(1-3N\right)\sin^{2}\;\rho }{\left(1-N\right)+\frac{1}{2}\left(3N-1\right)\;\sin^{2}\;\rho .}.$$ (13c)

This equation is the same as the one derived in [1] for the absorption of polarized light by molecules oriented in an ordered liquid crystal.

At this point it is desirable to test eqs (12a–f) for two limiting cases of *M* and *N* to check the correctness of the derivation.

(a) *Perfect ordering, N=1.0, M=1.0.* Under these conditions the six expressions reduce to

$$\bar{{\left(q_{x}^{V}\right)}_{0r}^{2}}/S^{V}\sigma_{0}Q=\cos^{2}\;\rho \;\cos^{2}\;\delta$$ (14a)

$$\bar{{\left(q_{y}^{V}\right)}_{0r}^{2}}/S^{V}\sigma_{0}Q=\bar{{\left(q_{z}^{V}\right)}_{0r}^{2}}/S^{V}\sigma_{0}Q=\frac{1}{2}\cos^{2}\;\rho \;\sin^{2}\;\delta$$ (14b, c)

$$\bar{{\left(q_{x}^{H}\right)}_{0r}^{2}}/S^{H}\sigma_{0}Q=\frac{1}{2}\cos^{2}\;\delta \;\sin^{2}\;\rho$$ (14d)

$$\bar{{\left(q_{y}^{H}\right)}_{0r}^{2}}/S^{H}\sigma_{0}Q=\frac{1}{8}\left\{3\left(\cos^{2}\;\rho -1\right)\;\left(\cos^{2}\;\delta -1\right)-2\;\sin^{2}\;\rho \;\sin^{2}\;\delta \;\cos^{2}\;v\right\}$$ (14e)

$$\bar{{\left(q_{z}^{H}\right)}_{0r}^{2}}/S^{H}\sigma_{0}Q=\frac{1}{8}\left\{\left(\cos^{2}\;\rho -1\right)\;\left(\cos^{2}\;\delta -1\right)+2\;\sin^{2}\;\rho \;\sin^{2}\;\delta \;\cos^{2}\;v\right\}$$ (14f)

These equations show the interplay of the three angles which define the molecule. If these equations are used to calculate the total flux emitted by the sample for the two types of excitation, the following are obtained,

$$\begin{array}{c} \bar{{\left(q^{V}\right)}_{0r}^{2}}\equiv \bar{{\left(q_{x}^{V}\right)}_{0r}^{2}}+\bar{{\left(q_{y}^{V}\right)}_{0r}^{2}}+\bar{{\left(q_{z}^{V}\right)}_{0r}^{2}}\\ =S^{V}\sigma_{0}Q\cos^{2}\;\rho , \end{array}$$ (15a)

$$\begin{array}{c} \bar{{\left(q^{H}\right)}_{0r}^{2}}\equiv \bar{{\left(q_{x}^{H}\right)}_{0r}^{2}}+\bar{{\left(q_{y}^{H}\right)}_{0r}^{2}}+\bar{{\left(q_{z}^{H}\right)}_{0r}^{2}}\\ =S^{H}\sigma_{0}Q\frac{1}{2}\sin^{2}\;\rho . \end{array}$$ (15b)

These equations show the correct dependence on *ρ* and that the dependence on δ and *ν* drops out. Thus if *ρ* = 0*°* (absorption dipole oscillator along the long molecular axis), vertically polarized light is absorbed since all the long molecular axes are aligned in the vertical direction (parallel to electric field) and horizontally polarized light is not absorbed.

(b) *Random distribution, N = 1/3, M = 1*/5. Under these conditions eqs (12a–f) reduce to

$$\begin{array}{c} \bar{{\left(q_{x_{r}}^{V}\right)}^{2}}/S^{V}\sigma_{0}Q=\bar{{\left(q_{z_{r}}^{H}\right)}^{2}}/S^{H}\sigma_{0}=\frac{1}{15}\left\{2\;\cos^{2}\;\rho \;\cos^{2}\;\delta +2\;\sin^{2}\;\rho \;\sin^{2}\;\delta \;\cos^{2}v+4\;\cos\;\rho \;\cos\;\delta \;\sin\;\rho \;\sin\;\delta \;\cos\;v+1\right\}\\ =\frac{1}{15}\left(2\;\cos^{2}\theta +1\right) \end{array}$$ (16a)

$$\begin{array}{c} \bar{{\left(q_{y_{r}}^{V}\right)}^{2}}/S^{V}\sigma_{0}Q=\bar{{\left(q_{z_{r}}^{V}\right)}^{2}}/S^{V}\sigma_{0}Q=\bar{{\left(q_{x_{r}}^{H}\right)}^{2}}/S^{H}\sigma_{0}Q=\bar{{\left(q_{y_{r}}^{H}\right)}^{2}}/S^{H}\sigma_{0}Q\\ =\frac{1}{15}\left\{2-\left[\cos^{2}\;\rho \;\cos^{2}\;\delta +2\cos\;\rho \;\cos\;\delta \;\sin\;\rho \;\sin\;\delta \;\cos\;v+\sin^{2}\;\rho \;\sin^{2}\;\delta \;\cos^{2}\;v\right]\right\}\\ =\frac{1}{15}\left(2-\cos^{2}\theta \right) \end{array}$$ (16b)

where eq (1c) has been used to express the results in terms of *θ.* These equations are the same as the ones obtained in a previous paper on the polarization of fluorescence of a random but frozen distribution of absorbing molecules [3]. Notice that information on *θ*, the angle between the absorption and emission dipole oscillators, is easily obtained when polarizers are placed in the beams. When eqs (16a, 16b) are used to calculate the total flux emitted by the sample for vertically and horizontally polarized excitation, one obtains

$$\begin{array}{l} {\left(q_{r}^{V}\right)}^{2}\equiv \bar{{\left(q_{x_{r}}^{V}\right)}^{2}}+\bar{{\left(q_{y_{r}}^{V}\right)}^{2}}+\bar{{\left(q_{z_{r}}^{V}\right)}^{2}}\\ \;=\frac{1}{3}S^{V}\sigma_{0}Q, \end{array}$$ (17a)

$$\begin{array}{l} {\left(q_{r}^{H}\right)}^{2}\equiv \bar{{\left(q_{x_{r}}^{H}\right)}^{2}}+\bar{{\left(q_{y_{r}}^{H}\right)}^{2}}+\bar{{\left(q_{z_{r}}^{H}\right)}^{2}}\\ \;=\frac{1}{3}S^{H}\sigma_{0}Q. \end{array}$$ (17b)

This result shows that the sample is an isotropic absorber and that there is no dependence on *ρ*, as there was in the case of perfect ordering shown by eqs (15a, b).

### 2.3. Application to the DPH-liquid crystal system

Previous studies have shown [1, 2] that the longest wavelength absorption band of DPH is due to a transition involving the fully conjugated molecule and that the absorption dipole oscillator lies along the long molecular axis; i.e., *ρ* = 0°. Also, the fluorescence emission dipole oscillator is almost parallel to the absorption dipole oscillator [1, 2], and therefore it also lies along the long molecular axis; i.e., δ = *θ = v* = 0°. Finally it has been shown that for DPH dissolved in the ordered liquid crystal mixture used here, the excited DPH molecules do not rotate at all during the time interval between absorption and emission [2]. With these conditions, the six expressions given by eqs (12a–f) reduce to

$$\bar{{\left(q_{x}^{V}\right)}^{2}}/S^{V}\sigma_{1}Q=M,$$ (18a)

$$\bar{{\left(q_{\gamma }^{V}\right)}^{2}}/S^{V}\sigma_{0}Q=\bar{{\left(q_{z}^{V}\right)}^{2}}/S^{V}\sigma_{0}Q=\bar{{\left(q_{x}^{H}\right)}^{2}}/S^{H}\sigma_{0}Q=\left(\frac{N-M}{2}\right),$$ (18b, c, d)

$$\bar{{\left(q_{\gamma }^{H}\right)}^{2}}/S^{H}\sigma_{0}Q=\left(\frac{1-2N+M}{8}\right)$$ (18e)

$$\bar{{\left(q_{z}^{H}\right)}^{2}}/S^{H}\sigma_{0}Q=3\left(\frac{1-2N+M}{8}\right).$$ (18f)

The fluorescence intensity readings which one obtains for the two viewing positions, 90° (rt angle) and 180° (straight through), of an ordered DPH sample can now be written with reference to eqs (8a–d) as

$$R_{V}^{V}\left(90{}^{\circ}\right)=R_{V}^{V}\left(180{}^{\circ}\right)=kS^{H}T_{H}\;FG\sigma_{0}Q\;M,$$ (19a)

$$R_{H}^{V}\left(90{}^{\circ}\right)=R_{H}^{V}\left(180{}^{\circ}\right)=kS^{H}T_{H}\;F\;\sigma_{0}Q\;\left(\frac{N-M}{2}\right),$$ (19b)

$$R_{V}^{H}\left(90{}^{\circ}\right)=R_{V}^{H}\left(180{}^{\circ}\right)=kS^{H}T_{H}\;G\;\sigma_{0}Q\;\left(\frac{N-M}{2}\right),$$ (19c)

$$R_{H}^{H}\left(90{}^{\circ}\right)=kS^{H}T_{H}\sigma_{0}Q\left(\frac{1-2N+M}{8}\right),$$ (19d)

$$R_{H}^{H}\left(180{}^{\circ}\right)=kS^{H}T_{H}\sigma_{0}Q\frac{3}{8}\left(1-2N+M\right),$$ (19e)

where *F ≡ S^V^/S^H^* is the polarization ratio of the exciting flux reaching the sample and *G ≡ T_V_/T_H_* is the polarization ratio of the emission detection system. As written, *F* and *G* are instrumental parameters and, with this liquid crystal system, can be determined easily since the unordered liquid crystal (i.e., no electric field applied to the sample) is cloudy and opaque, and acts as a light scatterer and depolarizer. Thus randomized fluorescence intensity readings, analogous to those given by eqs (19a–e), can be taken with the electric field off, and can be expressed as

$$R_{V}^{V}{\left(90{}^{\circ}\right)}_{r}=R_{V}^{V}{\left(180{}^{\circ}\right)}_{r}=k_{r}S^{H}T_{H}FG\sigma_{0}Q,$$ (20a)

$$R_{H}^{V}{\left(90{}^{\circ}\right)}_{r}=R_{H}^{V}{\left(180{}^{\circ}\right)}_{r}=k_{r}S^{H}T_{H}F\sigma_{0}Q,$$ (20b)

$$R_{V}^{H}{\left(90{}^{\circ}\right)}_{r}=R_{V}^{H}{\left(180{}^{\circ}\right)}_{r}=k_{r}S^{H}T_{H}G\sigma_{0}Q,$$ (20c)

$$R_{H}^{H}{\left(90{}^{\circ}\right)}_{r}=R_{H}^{H}{\left(180{}^{\circ}\right)}_{r}=k_{r}S^{H}T_{H}\sigma_{0}Q.$$ (20d)

where *k_r_* differs from *k* due to the scattering of the unordered crystal.

Taking the following ratios

$$F=\frac{R_{V}^{V}{\left(90{}^{\circ}\right)}_{r}}{R_{V}^{H}{\left(90{}^{\circ}\right)}_{r}}=\frac{R_{H}^{V}{\left(90{}^{\circ}\right)}_{r}}{R_{H}^{H}{\left(90{}^{\circ}\right)}_{r}}=\frac{R_{V}^{V}{\left(180{}^{\circ}\right)}_{r}}{R_{V}^{H}{\left(180{}^{\circ}\right)}_{r}}=\frac{R_{H}^{V}{\left(180{}^{\circ}\right)}_{r}}{R_{H}^{H}{\left(180{}^{\circ}\right)}_{r}},$$ (21a)

$$G=\frac{R_{V}^{V}{\left(90{}^{\circ}\right)}_{r}}{R_{H}^{V}{\left(90{}^{\circ}\right)}_{r}}=\frac{R_{V}^{H}{\left(90{}^{\circ}\right)}_{r}}{R_{H}^{H}{\left(90{}^{\circ}\right)}_{r}}=\frac{R_{V}^{V}{\left(180{}^{\circ}\right)}_{r}}{R_{H}^{V}{\left(180{}^{\circ}\right)}_{r}}=\frac{R_{V}^{H}{\left(180{}^{\circ}\right)}_{r}}{R_{H}^{H}{\left(180{}^{\circ}\right)}_{r}},$$ (21b)

gives estimates of *F* and *G* for the two different viewing positions. If the sample is a point source emitter, and if the two viewing positions, 90° and 180°, are equivalent in terms of the solid angle of emission collected, then not only should the estimates of *F* and *G* at each viewing position agree with one another but also the individual readings, such as $R_{V}^{V}{\left(90{}^{\circ}\right)}_{r}$ and $R_{V}^{V}{\left(180{}^{\circ}\right)}_{r}$, should agree. These two conditions, as well as the assumption that the solvent does not absorb any of the exciting light are inherent in the derivation of the equations and, as will be shown, must be modified if they do not apply. Nevertheless, corresponding readings of the two sets of data (field on and field off) can always be divided to give

$$VV\left(90{}^{\circ}\right)=VV\left(180{}^{\circ}\right)=k^{\prime }M,$$ (22a)

$$VH\left(90{}^{\circ}\right)=VH\left(180{}^{\circ}\right)=k^{\prime }\left(\frac{N-M}{2}\right),$$ (22b)

$$HV\left(90{}^{\circ}\right)=HV\left(180{}^{\circ}\right)=k^{\prime }\left(\frac{N-M}{2}\right),$$ (22c)

$$HH\left(90{}^{\circ}\right)=k^{\prime }\left(\frac{1-2N+M}{8}\right),$$ (22d)

$$HH\left(180{}^{\circ}\right)=k^{\prime }\frac{3}{8}\left(1-2N+M\right),$$ (22e)

where *k*′ = *k/k_r_*, and $VV\left(90{}^{\circ}\right)=R_{V}^{V}\left(90{}^{\circ}\right)/R_{V}^{V}{\left(90{}^{\circ}\right)}_{r}$, etc. The first letter of this new notation refers to the mode of the excitation polarizer and the second to the emission polarizer. Ratios of these equations will give information on the values of *N* and *M* that the sample exhibits.

## 3. Experimental Data [6]

Experimental data were taken using two different spectrofluorimeters. Preliminary data were taken using the Nottingham spectrofluorimeter [7], which is a right-angle viewing instrument. Polacoat 105 UV-visible polarizers were used in both the excitation and emission beams. The sample used was a 7.2 × 10^−5^ g DPH/1.055g L.C.M. mixture where the L.C.M. was also a 1.95/1 by weight mixture of cholesterol chloride to cholesterol laurate [5]. The sample cell consisted of a piece of 7.5 mm diameter Spectrosil tubing with a stationary bottom electrode and an adjustable top electrode with the liquid crystal mixture sandwiched between. A detailed description of this cell is given in [2]. The cylindrical cell was enclosed in a three-windowed aluminum heating block. The nominal electrode gap used was ~ 4 mm and the temperature of the system was kept at *T*_nem_ = 30 ± 1 °C.

Additional data, allowing a test of the above theory, were taken using the new NBS goniospectro-fluorimeter [8]. This versatile instrument allows viewing of a cylindrical sample from any arbitrary viewing angle from about 20° to 180°. However, the three-windowed aluminum heating block used in study limited the viewing of the liquid crystal sample cell to the 90° and 180° viewing positions. The sample cell used here was similar to the one above except that 3 mm diameter tubing and electrodes were used. Also, a lower concentration of DPH in the L.C.M. was used −1.2 × 10^−7^ DPH/1.104g L.C.M. This was done to approximate a point source sample by making the cell physically small, and also by keeping the light intensity constant as it propagated through the cell. Glan-Taylor polarizers were used with this instrument with a Corning 7–54 filter placed in the excitation beam and a 1 cm path length cut-off filter (saturated solution of NaNO_2_ in water) in the emission beam. The filter combination allowed viewing of the sample cell at 180° (straight through) by reducing the amount of scattered and/or nonabsorbed exciting light which reached the detector. Again, the electrode gap used was 4 mm and the temperature of the cell was kept at 30 ± 1 °C.

## 4. Results and Discussion

### 4.1. Degree of Polarization as a Function of Applied Electric Field Strength

**Figure 3:**
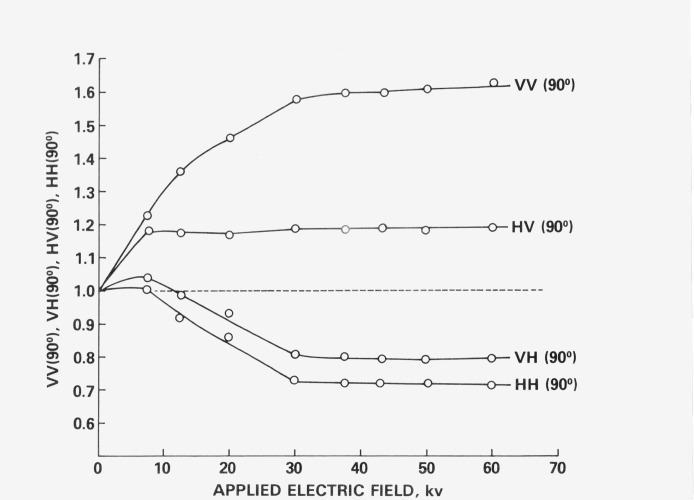
Degree of polarization of an ordered sample as a function of applied electric field strength for the four sets of orthogonal polarizer settings.

Using the Nottingham spectrofluorimeter and sample, the excitation wavelength was set at λ_ex_ = 370 nm and the emission wavelength at λ_em_ = 425 nm. Without the electric field applied, the four readings $R_{V}^{V}{\left(90{}^{\circ}\right)}_{r}$, $R_{H}^{V}{\left(90{}^{\circ}\right)}_{r}$, $R_{V}^{H}{\left(90{}^{\circ}\right)}_{r}$ and $R_{H}^{H}{\left(90{}^{\circ}\right)}_{r}$ were taken. Then the electric field was applied in steps up to 60 kV/cm and the four readings $R_{V}^{V}(90{}^{\circ})$, $R_{H}^{V}(90{}^{\circ})$, $R_{V}^{H}(90{}^{\circ})$ and $R_{H}^{H}(90{}^{\circ})$ were taken at each voltage. These readings were then divided by the ones with the field off to give *VV*(90°), *VH*(90°), *HV*(90°) and *HH*(90°) for each voltage setting. These data are plotted in figure 3 against applied voltage and show that the readings reach steady state values after 30 kV/cm, thus showing that single domain conditions are achieved, and that as long as the voltage is above 30 kV/cm the signal level will remain constant and will be unaffected by small drifts in the electric field strength. Therefore, all further experiments were performed with voltages of 40 kV/cm.

### 4.2. Polarization as a Function of Wavelength

Again using the Nottingham spectrofluorimeter and sample, the emission wavelength was set at λ_em_ = 425 nm and the four readings with the electric field on $-R_{V}^{V}(90{}^{\circ})$, $R_{H}^{V}(90{}^{\circ})$, $R_{V}^{H}(90{}^{\circ})$ and $R_{H}^{H}(90{}^{\circ})$—were taken as a function of excitation wavelength from 245 to 400 nm. Ratios of these readings were then taken with reference to eqs (19a–e) to give

$$R_{V}^{V}(90{}^{\circ})/R_{H}^{V}(90{}^{\circ})=G\left(\frac{2M}{N-M}\right),$$ (23a)

$$R_{V}^{H}(90{}^{\circ})/R_{H}^{H}(90{}^{\circ})=G4\left(\frac{N-M}{1-2N+M}\right).$$ (23b)

**Figure 4:**
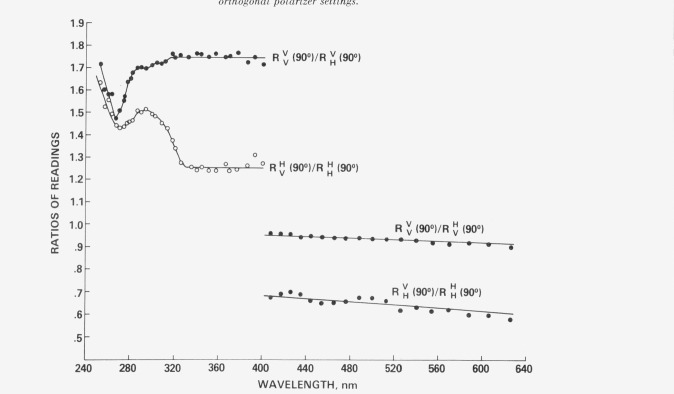
Degree of polarization of an ordered sample as a function of excitation and emission wavelength.

These particular ratios were calculated so as to eliminate the parameter *F*, which is a function of excitation wavelength. Here, *G* is constant since the emission wavelength does not change. The two ratios are plotted in figure 4 and show that with excitation into the longest wavelength absorption band, determinations of *N* and *M* are wavelength independent thus indicating that there is indeed only one absorption dipole absorbing in this region. The two ratios become wavelength dependent at shorter wavelengths due to increased solvent absorption, and also due to the appearance of other transitions in the absorption spectrum which have different angles between their absorption dipole oscillators and the long molecular axis [1]. Thus λ_ex_ = 370 nm was chosen at the optimum excitation wavelength.

With λ_ex_ = 370 nm, the four readings $R_{V}^{V}(90{}^{\circ})$, $R_{H}^{V}(90{}^{\circ})$, $R_{V}^{H}(90{}^{\circ})$ and $R_{H}^{H}(90{}^{\circ})$ were taken as a function of emission wavelength from 408 to 625 nm. The ratios

$$R_{V}^{V}(90{}^{\circ})/R_{V}^{H}(90{}^{\circ})=F\left(\frac{2M}{N-M}\right)$$ (23c)

$$R_{H}^{V}(90{}^{\circ})/R_{H}^{H}(90{}^{\circ})=4F\left(\frac{N-M}{1-2N+M}\right)$$ (23d)

were then calculated and are also shown in figure 4. The nearly horizontal straight lines show that the degree of polarization of the fluorescence emission is also independent of emission wavelength, as one would expect, and that fluorescence occurs from only one emission dipole oscillator.

### 4.3. Quantitative Test of Theoretical Model

Using the NBS spectrofluorimeter and sample with the excitation wavelength set at λ_ex_=370 nm and with the emission wavelength set at λ_em_ = 465 nm, fluorescence intensity readings were taken with and without the applied electric field at the two viewing positions, *α* = 90° and 180°. These data are shown in table 1. The readings with the electric field off were divided according to eqs (21a, b) to give the estimates of *F* and *G* shown in table 2. The agreement of the individual field off readings in table 1 for the two viewing positions, 90° and 180°, show that the sample is essentially a point source. The agreement of the values of *F* in table 2 show that the unordered liquid crystal is acting as a depolarizer. The values of *G* in table 2 agree for a given viewing angle but change when going from the 90° to the 180° viewing position. This is not surprising since the emission is being detected through two different windows in the heating block.

When the electric field is turned on, the individual polarization readings change from the values they had with the electric field off. However, the data in table 1 indicate that the two viewing positions are no longer equivalent since according to eqs (19a–e) only the *R^H^* reading should have changed in going from 90° to 180° viewing, and then only by a factor of 3 instead of 3.43. Even if the assumed model for the DPH dipoles is in error the $R_{V}^{V}(90{}^{\circ})$ and $R_{H}^{V}(90{}^{\circ})$ readings should still equal the $R_{V}^{V}(180{}^{\circ})$ and $R_{H}^{V}(180{}^{\circ})$ readings respectively. Therefore, this discrepancy is attributed to the fact that, with the field on, the sample is no longer a point source. There are two reasons for this. First, the ordered liquid crystal mixture is strongly birefrigent and, therefore, not equivalent to the unordered sample. Thus, the *G* correction factor estimated from the latter is not quite correct. Secondly, the liquid crystal solvent has been shown to have its own dichroic ratio for absorption when the electric field is on and, therefore, will modify the polarization ratio *F* of the exciting radiation in wavelength regions of solvent absorption. Thus, the ratio of the field on to field off data as given by eqs (22a–e) should really be written as,

$$VV(\alpha )=k_{\alpha }(1-e^{-A(V)})T_{V}^{\prime }\;M$$ (24a)

$$VH(\alpha )=k_{\alpha }(1-e^{-A(V)})T_{H}^{\prime }\;\left(\frac{N-M}{2}\right)$$ (24b)

$$HV(\alpha )=k_{\alpha }(1-e^{-A(H)})T_{V}^{\prime }\;\left(\frac{N-M}{2}\right)$$ (24c)

$$HH(90{}^{\circ})=k_{90^{{}^{\circ}}}(1-e^{-A(H)})T_{H}^{\prime }\;\left(\frac{1-2N+M}{8}\right)$$ (24d)

$$HH(180{}^{\circ})=k_{180^{{}^{\circ}}}(1-e^{-A(H)})T_{H}^{\prime }\frac{3}{8}\;\left(1-2N+M\right)$$ (24e)

where *α* = 90° or 180°, *A*(*V*) and *A*(*H*) are the absorbances for vertically and horizontally polarized exciting light, respectively, $T_{V}^{\prime }$ is another responsivity factor for vertically polarized emission due to the birefrigence of the sample, and $T_{H}^{\prime }$ is the horizontal responsitivity. The proportionality constants *k*_90°_ and *k*_180°_ differ because the sample is no longer a point source.

The best way to eliminate any effect due to the solvent absorption is to obtain ratios only from the readings which have constant excitation polarization and constant viewing angle. Thus, we define the ratios

$$\frac{VV(90{}^{\circ})}{VH(90{}^{\circ})}=\frac{2M}{N-M}G^{\prime }$$ (25a)

$$\frac{HV(90{}^{\circ})}{HH(90{}^{\circ})}=4\left(\frac{N-M}{1-2N+M}\right)G^{\prime }$$ (25b)

$$\frac{VV(180{}^{\circ})}{VH(180{}^{\circ})}=\frac{2M}{N-M}G^{\prime }$$ (25c)

$$\frac{HV(180{}^{\circ})}{HH(180{}^{\circ})}=\frac{4}{3}\left(\frac{N-M}{1-2N+M}\right)G^{\prime }$$ (25d)

where $G^{\prime }=T_{V}^{\prime }{/}_{H}{\;}^{\prime }$.

### 4.4. Determination of G′

The polarization ratio, *G*′ due to the birefringence of the ordered liquid crystal solvent was determined experimentally by removing the filter in the excitation beam and setting the excitation monochromator wavelength to λ*_ex_* = 465 nm to match the fluorescence emission wavelength. With the emission detection system set at the 180° viewing position the 465 nm flux was monitored as it propagated through the liquid crystal cell. The four intensity readings $-R_{V}^{V^{\prime }}(180{}^{\circ})$, $R_{H}^{V^{\prime }}(180{}^{\circ})$, $R_{V}^{H^{\prime }}(180{}^{\circ})$ and $R_{H}^{H^{\prime }}(180{}^{\circ})$ –were also taken with and without the applied electric field (see table 1). Since neither the liquid crystal solvent nor the DPH absorb at this wavelength the effect of the birefringence can now be determined directly. The readings with the electric field off were then divided according to eqs (21a) and (21b) to give estimates of *G* and *F* (465 nm) which are then given in table 2. The estimate of *G* agrees with the previous estimates derived from the fluorescence intensity readings, but the value of *F* (465 nm) differs from the value of *F* (370 nm) determined previously. This is to be expected since *F* is a function of excitation wavelength. The data obtained with the field on (table 1, bottom) show that the $R_{H}^{V^{\prime }}(180{}^{\circ})$ and $R_{V}^{H^{\prime }}(180{}^{\circ})$ readings are small compared to the $R_{V}^{V^{\prime }}(180{}^{\circ})$ and $R_{H}^{H^{\prime }}(180{}^{\circ})$ readings, thus showing that the ordered liquid crystal is not depolarizing the light as it propagates through the solution and any effect is thus due to the way the emission detection system views the birefringent sample. Thus the ratio of the $R_{V}^{V^{\prime }}(180{}^{\circ})$ and $R_{H}^{H^{\prime }}(180{}^{\circ})$ readings can be written as,

$$\frac{R_{V}^{V^{\prime }}(180{}^{\circ})}{R_{H}^{H^{\prime }}(180{}^{\circ})}=\frac{S{\;}^{V}T_{V}^{\prime }T_{V}}{S{\;}^{H}T_{H}^{\prime }T_{H}}=F(465\;\text{nm})G^{\prime }G.$$ (26)

Solving eq (26) for *G'* using $R_{V}^{V}(180{}^{\circ})=0.2113$, $R_{H}^{H}(180{}^{\circ})=0.9276$, *F* 465 nm) = 0.5845 and *G* = 0.6275 gives a value of *G'* = 0.6210.

### 4.5. Determination of *M* and *N*

With *G'* determined, eqs (25a–d) can be rearranged to give

$$\frac{N}{M}=2\frac{VH(90{}^{\circ})}{VV(90{}^{\circ})}G^{\prime }+1,$$ (27a)

$$\frac{1}{M}=4\frac{HH(90{}^{\circ})}{HV(90{}^{\circ})}G^{\prime }\left(\frac{N}{M}-1\right)+2\frac{N}{M}-1,$$ (27b)

$$\frac{N}{M}=2\frac{VH(180{}^{\circ})}{VV(180{}^{\circ})}G^{\prime }+1,$$ (27c)

$$\frac{1}{M}=\frac{4}{3}\frac{HH(180{}^{\circ})}{HV(180{}^{\circ})}G^{\prime }\left(\frac{N}{M}-1\right)+2\frac{N}{M}-1.$$ (27d)

Equations (27a) and (27b) can now be solved simultaneously, using the 90° data of the “Ratio” column in table 1 to obtain estimates of *M* and *N*. Likewise, eqs (27c) and (27d) with the 180° data in the same column will give estimates of *M* and *N.* These are tabulated in table 3a along with a value of *κ* for each estimate from the curves in figure 2.

In order to check the precision of these results, the measurements were repeated. The electric field was turned off and it was found that after a few minutes the sample returned reversibly to its initial unordered state, as the polarization readings were the same as those obtained before the electric field had been applied the first time. Then a second set of data was taken and processed in the same manner as before. The final results for *N, M*, and *κ* are given in table 3b, showing that for each viewing angle the *M* and *N* values of table 3a were reproduced to 0.01. Thus the differences of the *M* values for 90° and 180° viewing are within the experimental uncertainty for both sets of data. The values for *N* obtained for 180° viewing are, however, consistently higher than the 90° values by about three times the experimental reproducibility. This was also observed from preliminary data taken when the experiment was first being set up. Also the average value of *N* = 0.55 from the data in table 3 is lower than the value of 0.633 obtained previously [1] from a study of the absorption of polarized light by DPH molecules dissolved in the same L.C.M. This may be due to the fact that in this study the cylindrical sample cell has more bulk solution than the thin rectangular cell used in the absorption experiment. Another possibility is that the model of the DPH molecule may not be quite correct as far as the angles of the different dipoles oscillators are concerned. Small variations in angles or molecule movement could cause large changes in the magnitudes of *N* and *M* as well as differences of their values as a function of viewing angle as can be seen by examination of eqs (12a–f). The differences in the values of *κ* as determined from *N* data compared to *M* data can be attributed to the type of distribution function used if indeed the average values of *N* and *M* are correct.

## Figures and Tables

**Table 1:** Experimental readings taken with and without the applied electric field for the determination of the degree of polarization of the fluorescence emission and for the determination of the correction factor due to the birefringence of the ordered liquid crystal solvent.

λ*_ex_* = 370 nm λ*_em_* = 465 nm	Field OFF		Field ON		$\text{Ratio}\frac{\text{ON}}{\text{OFF}}$	

	Volts		Volts		90°	180°
	90°	180°	90°	180°		
$R_{V}^{V}$	0.05295	0.05469	0.08422	0.15763	1.5906	2.8822
$R_{H}^{V}$	.09032	.08732	.06125	.11744	0.67814	1.3449
$R_{V}^{H}$	.09759	.10123	.08657	.15156	.88708	1.4972
$R_{H}^{H}$	.16486	.16176	.09116	.31235	.55295	1.9310
λ*_ex_* = 465 nm						
λ*_em_* = 465 nm						
$R_{V}^{V^{\prime }}$	–	.31083	–	.21128		
$R_{H}^{V^{\prime }}$	–	.49496	–	.01487		
$R_{V}^{H^{\prime }}$	–	.53113	–	.02222		
$R_{H}^{H^{\prime }}$	–	.84711	–	.92759		

**Table 2:** Table of instrumental polarization correction factors obtained from the data taken with the electric field off.

	λ*_ex_* = 370, λ*_em_* = 465		λ*_ex_* = 465, λ*_em_* = 465
	90°	180°	180°
			
$F=\frac{R_{V}^{V}{\left(\alpha \right)}_{r}}{R_{V}^{H}{\left(\alpha \right)}_{r}}$	0.543	0.540	0.585
$=\frac{R_{H}^{V}{\left(\alpha \right)}_{r}}{R_{H}^{H}{\left(\alpha \right)}_{r}}$	.548	.540	.584
$G=\frac{R_{V}^{V}{\left(\alpha \right)}_{r}}{R_{H}^{V}{\left(\alpha \right)}_{r}}$	.586	.626	.628
$=\frac{R_{V}^{H}{\left(\alpha \right)}_{r}}{R_{H}^{H}{\left(\alpha \right)}_{r}}$	.592	.626	.627

**Table 3:** Calculated values of the orientation terms *N* and *M* evaluated from experimental data taken at two different viewing positions of the liquid crystal-DPH sample. The value of *κ* for each *N* and *M* is determined from the curves in figure 2.

(a) First Experiment				
	*M*	*κ*	*N*	*κ*
				
90° Data	0.35	2.1	0.53	2.4
180° Data	.36	2.2	.57	2.9

(b) Second Experiment				
	*M*	*κ*	*N*	*κ*
				
90° Data	0.34	2.0	0.53	2.4
180° Data	.35	2.1	.56	2.8

## Footnotes

1 Figures in brackets indicate the literature references at the end of this paper.
